# Auger Electrons Constructed Active Sites on Nanocatalysts for Catalytic Internal Radiotherapy

**DOI:** 10.1002/advs.201903585

**Published:** 2020-04-06

**Authors:** Weiwei Su, Han Wang, Tao Wang, Xiao Li, Zhongmin Tang, Shuai Zhao, Meng Zhang, Danni Li, Xingwu Jiang, Teng Gong, Wei Yang, Changjing Zuo, Yelin Wu, Wenbo Bu

**Affiliations:** ^1^ Department of Nuclear Medicine Changhai Hospital Naval Medical University Shanghai 200433 P. R. China; ^2^ University of Chinese Academy of Sciences Beijing 100049 P. R. China; ^3^ State Key Laboratory of High Performance Ceramics and Superfine Microstructures Shanghai Institute of Ceramics Chinese Academy of Sciences Shanghai 200050 P. R. China; ^4^ Tongji University Cancer Center Shanghai Tenth People's Hospital Tongji University School of Medicine Shanghai 200072 P. R. China; ^5^ Shanghai Key Laboratory of Green Chemistry and Chemical Processes School of Chemistry and Molecular Engineering East China Normal University Shanghai 200062 P. R. China

**Keywords:** active sites, auger Electrons, I‐125, internal radiotherapy, nanocatalysts, titanium dioxide

## Abstract

Excess electrons play important roles for the construction of superficial active sites on nanocatalysts. However, providing excess electrons to nanocatalysts in vivo is still a challenge, which limits the applications of nanocatalysts in biomedicine. Herein, auger electrons (AEs) emitted from radionuclide 125 (^125^I) are used in situ to construct active sites in a nanocatalyst (TiO_2_) and the application of this method is further extended to cancer catalytic internal radiotherapy (CIRT). The obtained ^125^I‐TiO_2_ nanoparticles first construct superficial Ti^3+^ active sites via the reaction between Ti^4+^ and AEs. Then Ti^3+^ stretches and weakens the O—H bond of the absorbed H_2_O, thus enhancing the radiolysis of H_2_O molecules and generating hydroxyl radicals (•OH). All in vitro and in vivo results demonstrate a good CIRT performance. These findings will broaden the application of radionuclides and introduce new perspectives to nanomedicine.

## Graphical abstract

A simple and highly efficient catalytic internal radiotherapy (CIRT) is developed by ^125^I‐labeled TiO_2_ nanoparticles. Auger electrons from ^125^I react with Ti^4+^ in TiO_2_ and construct superficial active sites of Ti^3+^. The generated Ti^3+^ decreases the energy barriers of H_2_O and contributes to the generation of •OH upon γ‐rays irradiation, thus enhancing the antitumor effect of IRT.

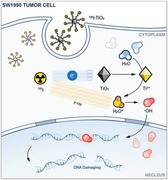

Nanocatalysts have been widely used in the area of biomedicine, such as cancer therapy, antibacterial treatment, and biomolecules detection, because of their high surface activity.^[^ ^1^, ^2^, ^3^ ^]^ Many published reports have shown that excess electrons (e.g., photoinduced electrons) play an important role in the surface chemical activity of nanocatalysts.^[^ ^4^, ^5^, ^6^ ^]^ Generally, excess electrons are captured by superficial metal atoms to form active sites with localized charge imbalance. Then, these active sites can deform certain molecules, stretch chemical bonds, and reduce the energy barriers of chemical reactions. Hence, providing excess electrons to the surface of nanocatalysts is important for improving the nanocatalyst performance. However, hardly few methods exist for the generation of excess electrons in biological tissue, which inhibits the application of nanocatalysts in tumor treatment.

Internal radiotherapy (IRT) is a common treatment method for suppressing tumor growth.^[^ ^7^, ^8^ ^]^ Some radionuclides, such as ^90^Y, ^125^I, ^131^I, and ^188^Re, can emit electrons in situ and are thus usually implanted into tumors for IRT.^[^ ^9^, ^10^, ^11^, ^12^ ^]^ Among these radionuclides, ^125^I can emit low‐energy (10^0^–10^3^ eV) auger electrons (AEs) via internal conversion with a production of 24.9 AEs per decay, which can be deposited on nanocatalysts more easily than the high‐energy (≈10^6^ eV) β‐rays emitted from other radionuclides.^[^ ^13^ ^]^ Hence, ^125^I has the potential to inject excess electrons onto nanocatalysts surface and construct active sites for further applications in vivo. On the other hand, in IRT, ionizing radiation (primarily β‐ and γ‐rays) emitted from radionuclides induces radiolysis of H_2_O to generate hydroxyl radicals (•OH), which can efficiently kill cancer cells.^[^ ^14^, ^15^, ^16^ ^]^ However, the strong O—H bond of H_2_O molecule limits the yield of •OH induced by radionuclides, and especially for ^125^I, which emits low‐energy AEs and γ‐rays (35 keV).^[^ ^17^, ^18^ ^]^ Fortunately, as mentioned above, the combination of nanocatalysts and AEs emitted from ^125^I will construct superficial active sites, which can stretch molecules and decrease the bond energy for H_2_O activation, thus facilitating the occurring of H_2_O radiolysis. Hence, ^125^I‐labeled nanocatalysts have the potential to increase the yield of •OH for enhanced IRT.

Titanium dioxide (TiO_2_) is a traditional and typical nanocatalyst with high stability and low toxicity, which is suitable for applications in vivo.^[^ ^19^, ^20^, ^21^, ^22^ ^]^ Herein, we developed a method of injecting AEs to nanocatalysts by synthesizing ^125^I‐labeled TiO_2_ nanoparticles (^125^I‐TiO_2_ NPs) and explored the performance of ^125^I‐TiO_2_ NPs for cancer catalytic internal radiotherapy (CIRT), as illustrated in **Scheme** **1**. First, AEs emitted from ^125^I arrive on the surface of TiO_2_ and induce the formation of Ti^3+^. Then, Ti^3+^ absorbs and deforms H_2_O, resulting in the decreased O—H bond energy. Finally, upon irradiation by the γ‐rays emitted from ^125^I, the activated H_2_O is more easily converted to •OH compared to the unabsorbed H_2_O, leading to an enhanced effect of IRT. This strategy as CIRT will bring more chances for cancer therapy. Moreover, the method of constructing active sites by ^125^I will also widen the applications of radionuclides and nanocatalysts and introduce new perspectives to the area of nanocatalytic medicine.

**Scheme 1:**
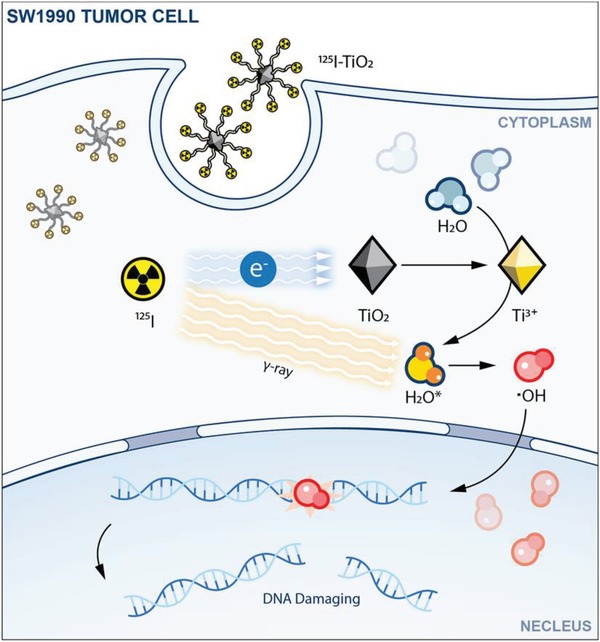
Schematic illustration for the mechanism of ^125^I‐TiO_2_‐induced CIRT.

The synthesis process of ^125^I‐TiO_2_ is illustrated in **Figure** **1**a. First, TiO_2_ NPs coated with oleylamine and oleic acid (TiO_2_‐OA) were synthesized according to a typical solvothermal method.^[^ ^23^, ^24^ ^]^ In Figure 1b, transmission electron microscope (TEM) image showed that TiO_2_‐OA were in good uniformity with rhombus‐shaped morphology, and the average particle diameter was 11.78 ± 2.23 nm in length and 3.91 ± 0.56 nm in width. High‐resolution TEM (HRTEM) image revealed a lattice fringe width of 0.35 nm, corresponding to the anatase (confirmed by X‐ray diffraction (XRD) patterns in Figure 1c) (101) crystal face. Subsequently, TiO_2_‐OA were modified with citric acid (TiO_2_‐COOH) for well water solubility and to prepare for subsequent modification. Finally, TiO_2_‐COOH were conjugated with tyramine (TiO_2_‐tyr) for labeling of ^125^I. Successful synthesis of TiO_2_‐COOH and TiO_2_‐tyr was confirmed by the presence of characteristic peaks (O—H and amide, respectively) in Fourier transform infrared spectroscopy (FT‐IR) spectra (Figure 1d).^[^ ^25^ ^]^ The absorption peak changed little in ultraviolet‐visible (UV–vis) spectra (Figure S1, Supporting Information), indicating that TiO_2_ remained stable after modification. Obviously, compared with TiO_2_‐COOH, the conjugated tyramine restrained the ionization of carboxyls, leading to a decreased Zeta potential (Figure S2, Supporting Information) and an increased hydrodynamic size (Figure 1e) of TiO_2_‐tyr. Finally, ^125^I was labeled to TiO_2_‐tyr via a classical Iodogen‐catalyzed method to obtain the final product of ^125^I‐TiO_2_.^[^ ^26^ ^]^ The initial radiochemical purity of ^125^I‐TiO_2_ was 93.43% and remained above 88% during a 24‐h incubation with 0.1% fetal bovine serum in phosphate buffer solution, signifying a successful and stable radiolabeling (Figure 1f and Figure S3, Supporting Information).

**Figure 1:**
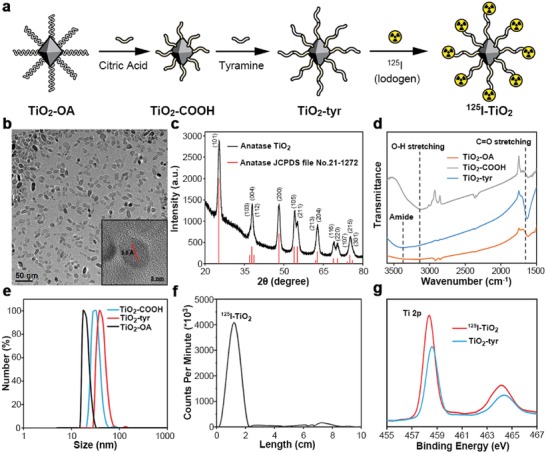
Synthesis and characterization of ^125^I‐TiO_2_. a) Synthesis process of ^125^I‐TiO_2_. b) TEM image of TiO_2_‐OA (inset: HRTEM image of TiO_2_‐OA). c) XRD pattern of TiO_2_‐OA. d) FT‐IR spectra of TiO_2_‐OA, TiO_2_‐COOH, and TiO_2_‐tyr. e) Hydrodynamic radius of TiO_2_‐OA, TiO_2_‐COOH, and TiO_2_‐tyr. f) Radio thin‐layer chromatography analyzing the labeling rate of ^125^I‐TiO_2_. g) XPS spectra of TiO_2_‐tyr and ^125^I‐TiO_2_.

After obtaining ^125^I‐TiO_2_, we investigated the interaction between TiO_2_ and H_2_O. First, X‐ray photoelectron spectroscopy (XPS) showed that the binding energy of Ti 2p orbitals of ^125^I‐TiO_2_ decreased by 0.2 eV compared with that of TiO_2_‐tyr, indicating the existence of Ti^3+^ in ^125^I‐TiO_2_ (Figure 1g).^[^ ^27^ ^]^ Next, density functional theory (DFT) was used to simulate the O—H bond length under the influence of Ti^4+^ or Ti^3+^. **Figure** **2**a shows that the O—H bond length was 1.17 Å under the influence of Ti^4+^. In contrast, Ti^3+^ stretched the O—H bond to 1.48 Å (Figure 2b), signifying a decreased bond energy. Further, the energy barrier for converting H_2_O to •OH was calculated. As shown in Figure 2c,d, the required energy for H_2_O radiolysis under the influence of Ti^4+^ was 1.23 eV, while declined to 0.82 eV under the influence of Ti^3+^. Hence, the Ti^3+^ species in ^125^I‐TiO_2_ can reduce the O—H bond energy and energy barrier of H_2_O radiolysis, indicating the potential to increase the yield of •OH in IRT.

**Figure 2:**
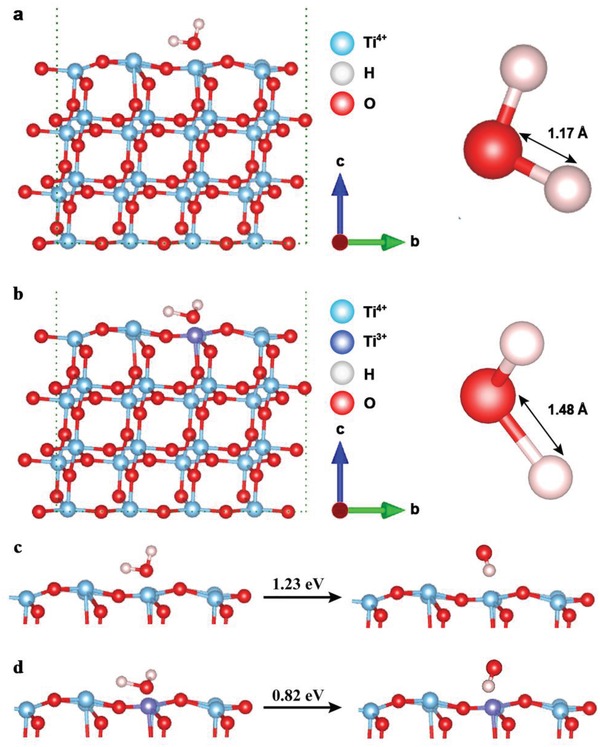
Simulated O—H bond length of H_2_O and the energy barrier for converting H_2_O to ·OH on the surface of TiO_2_ (anatase (101) face). a) O—H bond under the influence of Ti^4+^. b) O—H bond under the influence of Ti^3+^. c) Energy barrier of H_2_O radiolysis under the influence of Ti^4+^. d) Energy barrier of H_2_O radiolysis under the influence of Ti^3+^.

Pancreatic cancer usually induced a poor prognosis and short survival time for patients. Many clinical researches have reported that IRT was suitable for the treatment of pancreatic cancer.^[^ ^28^, ^29^, ^30^, ^31^, ^32^, ^33^, ^34^ ^]^ Hence, to investigate the effect of CIRT for pancreatic cancer, we adopted human pancreatic cancer (SW1990) cells for all experiments in vitro. First, as sufficient intracellular accumulation of NPs is a prerequisite for an effective treatment, the cellular uptake and intracellular distribution of TiO_2_‐tyr was visualized via biological TEM (bio‐TEM). As exhibited in Figure S4 in the Supporting Information, the amount of endocytosed TiO_2_‐tyr increased as the incubation time prolonged from 0.5 to 5 h. Next, we investigated the appropriate dosage of ^125^I and ^125^I‐TiO_2_ via the Cell Counting Kit 8 Assay. As illustrated in Figure S5 in the Supporting Information, ^125^I exhibited a negligible detrimental effect especially when the dosage was below 600 µCi mL^−1^. Compared to 600 µCi mL^−1^ of ^125^I (cell viability, 96.44%), ^125^I‐TiO_2_ with the equal dose (total mass of 144 µg mL^−1^, radiation dose of 600 µCi mL^−1^) presented obvious cytotoxicity (cell viability, 64.16%). Similar trends also appeared under other doses of radiation. As ^125^I or TiO_2_‐tyr alone exhibited little cytotoxicity to SW1990 cells (Figures S5 and S6, Supporting Information), the cell killing effect of ^125^I‐TiO_2_ should be attributed to the reaction between ^125^I and TiO_2_‐tyr. To fully explore the effect of ^125^I‐TiO_2_, the control group, ^125^I group (600 µCi mL^−1^), and ^125^I‐TiO_2_ group (total mass of 144 µg mL^−1^, radiation dose of 600 µCi mL^−1^) were adopted in this study for the following experiments.

It is widely known that radiation exerts biological effects mainly by inducing cell apoptosis and proliferative injury.^[^ ^35^, ^36^, ^37^ ^]^ Therefore, cell apoptosis was detected by terminal‐deoxynucleoitidyl Transferase Mediated Nick End Labeling (TUNEL) assays. As shown in **Figure** **3**a,b, ^125^I‐TiO_2_ triggered the most severe cell apoptosis (apoptosis rate, 37.48%), which was 13.82 and 37.62 times that of the ^125^I group and control group, respectively. Proliferative injury was first evaluated by the expression level of proliferating cell nuclear antigen (PCNA), which is a representative protein directly involved with the DNA replication.^[^ ^38^ ^]^ The expression of PCNA in ^125^I‐TiO_2_ group decreased by 61.05% and 73.78% compared to that in ^125^I group and control group, respectively (Figure 3c,d). Then, the colony formation assay, which is a classical and sensitive method for the visual evaluation of cell proliferation status,^[^ ^39^ ^]^ indicated that the cells in ^125^I‐TiO_2_ group had the lowest cell surviving fraction and cloning efficiency in a dose‐dependent manner (Figure 3e,f and Figure S7, Supporting Information). In addition, the expression of relevant regulatory proteins, including pro‐apoptotic Bax, antiapoptotic Bcl‐2, and proliferation index of Ki‐67, displayed similar tendencies consistent with above results (Figure S8, Supporting Information). In summary, the combination of TiO_2_ and ^125^I can induce increased cell apoptosis and suppressed cell proliferation compared to ^125^I alone.

**Figure 3:**
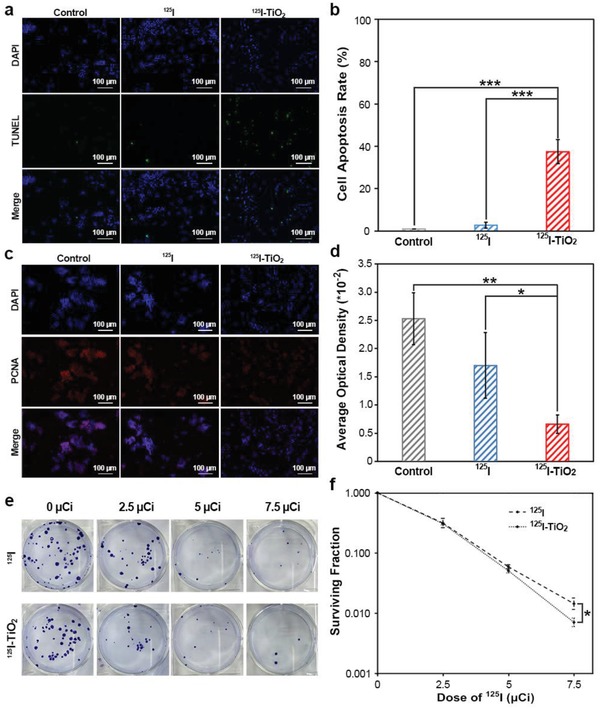
In vitro experiments for therapeutic evaluation. a) Immunofluorescence assay of TUNEL analyzing cell apoptosis induced by dulbecco's modified eagle medium (DMEM, control), ^125^I, and ^125^I‐TiO_2_. b) Quantitative comparison of cell apoptosis rate among groups (*n* = 3, mean ± s.d., one‐way analysis of variance (ANOVA) with least significant difference (LSD)‐t post hoc test, *P* < 0.0001 (^125^I‐TiO_2_ vs Control) and < 0.0001 (^125^I‐TiO_2_ vs ^125^I), ***P < 0.001). c) Immunofluorescence assay analyzing the cellular PCNA expression in different treatment groups including control, ^125^I, and ^125^I‐TiO_2_ group. d) Quantitative comparison of the expression level of PCNA among groups (*n* = 3, mean ± s.d., one‐way ANOVA with LSD‐t post hoc test, *P* = 0.0020 (^125^I‐TiO_2_ vs Control) and 0.0273 (^125^I‐TiO_2_ vs ^125^I), *P < 0.05, **P < 0.01). e) Colony formation assay analyzing cell proliferation after receiving incremental radiation doses of ^125^I and ^125^I‐TiO_2_. f) Curves of cell surviving fraction based on colony formation assay (*n* = 3, mean ± s.d., Mann–Whitney *U*‐test, *P* = 0.0463 (^125^I‐TiO_2_ vs ^125^I), *P < 0.05).

Cytotoxicity of radiation is usually attributed to the •OH‐induced DNA double‐strand breaks (DSBs).^[^ ^40^, ^41^ ^]^ As mentioned above, we designed ^125^I‐TiO_2_ to improve the yield of •OH in IRT. Hence, we initially measured the generation of intracellular •OH using a hydroxyphenyl fluorescein probe. As shown in **Figure** **4**a,b, the amount of •OH in ^125^I‐TiO_2_ group exhibited a substantial augmentation after 24 h of irradiation, which was 1.62 and 1.70 multiples of that in ^125^I and control groups, respectively. Then DNA DSBs were detected via γ‐H2AX immunofluorescence assay. According to Figure 4c,d, cancer cells treated by ^125^I‐TiO_2_ presented the most severe DNA DSBs (one γ‐H2AX foci represents one DNA DSB). These data proved that ^125^I‐TiO_2_ can enhance the generation of •OH, thereby exacerbating DNA DSBs, and ultimately cause cell apoptosis and proliferative injury.

**Figure 4:**
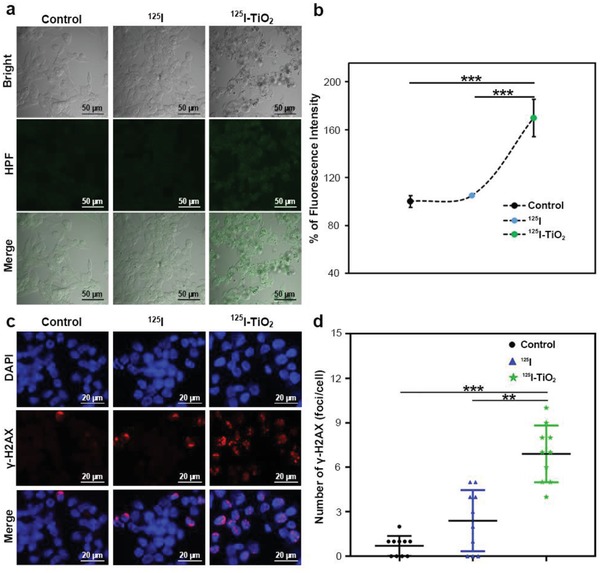
In vitro cell experiments exploring the mechanism for enhanced therapeutic effects. a) Fluorescence assay analyzing intracellular •OH generation after different treatments of DMEM (control), ^125^I and ^125^I‐TiO_2_. b) Quantitative comparison of •OH yield among groups (*n* = 3, mean ± s.d., one‐way ANOVA with LSD‐t post hoc test, *P* < 0. 0001 (^125^I‐TiO_2_ vs ^125^I) and = 0.0002 (^125^I‐TiO_2_ vs Control), ***P < 0.001). c) Immunofluorescence assay analyzing DNA DSBs by γ‐H2AX staining in DMEM (control), ^125^I, and ^125^I‐TiO_2_ groups. d) Quantitative comparison of DNA DSBs‐related red fluorescent foci of γ‐H2AX among groups (*n* = 10, mean ± s.d., Kruskal–Wallis one‐way ANOVA analysis, *P* = 0.002 (^125^I‐TiO_2_ vs ^125^I) and <0.001 (^125^I‐TiO_2_ vs Control), **P < 0.01, ***P < 0.001).

Encouraged by these in vitro experiments, we tested the effect of CIRT in SW1990 tumor‐xenografted mice. Three groups were divided with intratumoral injection of dulbecco's modified eagle medium (DMEM, control), free ^125^I (600 µCi of ^125^I per mouse) and ^125^I‐TiO_2_ (600 µCi of ^125^I corresponding to 144 µg of TiO_2_ per mouse), respectively. First, single‐photon emission computed tomography/computed tomography (SPECT/CT) scanning was performed. As shown in **Figure** **5**a, most of the injected ^125^I was excreted through the urinary system with only minor residuals drawn in the thyroid. In a sharp contrast, ^125^I‐TiO_2_ exhibited remarkably prolonged retention time in the tumor for more than 12 d, which indicated an excellent labeling stability and intratumoral retention ability of ^125^I‐TiO_2_ in vivo, thus benefiting for a long‐lasting irradiation for tumor.

**Figure 5:**
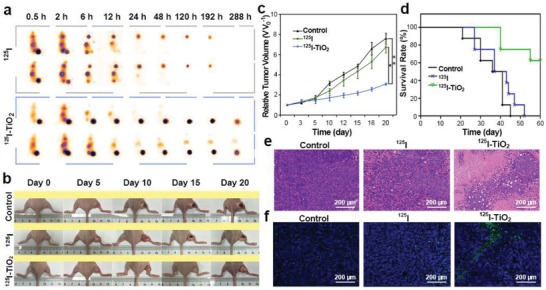
In vivo imaging and therapeutic assessment. a) Representative SPECT/CT images of SW1990 tumor‐bearing mice at different time points after intratumoral injection of ^125^I and ^125^I‐TiO_2_. b) Representative photographs of SW1990 tumor‐bearing mice for time‐course change of tumor size after different treatments including DMEM (control), ^125^I, and ^125^I‐TiO_2_. c) Tumor growth curves during the 20 d observation after different treatments by DMEM (control), ^125^I, and ^125^I‐TiO_2_. Relative tumor volume (*V V*_0_^−1^) was given by tumor volume (*V*) normalized to the initial value (*V*_0_) (*n* = 5, mean ± s.d., Kruskal–Wallis one‐way ANOVA analysis, *P* = 0.048 (^125^I‐TiO_2_ vs ^125^I) and 0.001 (^125^I‐TiO_2_ vs Control), *P < 0.05, **P < 0.01). d) Survival curves of mice after different treatments during an observation period of 60 d (*n* = 8). e) H&E staining of tumor sections harvested at day 20 post different treatments (original magnification 200 ×). f) Representative TUNEL staining of tumor slices collected at day 20 post treatments (original magnification 200 ×).

We further examined the curative effect of ^125^I‐TiO_2_ in vivo. As shown in Figure 5b,c and Figure S9 in the Supporting Information, after a 20 d treatment, tumors in control and ^125^I groups progressed to 7.58 ± 0.54 and 6.71 ± 0.55 times that of the original volumes, whereas tumors treated by ^125^I‐TiO_2_ were distinctly controlled with the relative volume percentages of 59.49% (for control group) and 54.21% (for ^125^I group), respectively. Correspondingly, the survival rate within a 60 d observation was significantly improved in ^125^I‐TiO_2_ group, during which all mice in ^125^I and control groups died out (Figure 5d). Moreover, all surviving mice had reasonably stable body weights during the observation period, indicating a negligible long‐term biotoxicity of all compositions (Figure S10, Supporting Information). The histopathologic results also supported the above findings. As expected, hematoxylin and eosin (H&E) staining (Figure 5e) and TUNEL (Figure 5f) revealed that the most severe tumor tissue destruction occurred after the tumors were exposed to ^125^I‐TiO_2_ rather than to ^125^I. According to immunohistochemical staining results, the expression of Ki‐67 (a cancer cell proliferation factor) was downregulated in ^125^I‐TiO_2_ group compared to that in ^125^I or control groups (Figure S11, Supporting Information), while the TNF‐α (a tumor necrosis factor) expressed the highest in ^125^I‐TiO_2_ group (Figure S12, Supporting Information). All these data proved the highly efficient anticancer ability of ^125^I‐TiO_2_.

In summary, we used ^125^I to inject electrons to TiO_2_ NPs for constructing active sites in vivo, and investigated its application for cancer CIRT. The obtained ^125^I‐TiO_2_ initially constructed Ti^3+^ species via the reaction between Ti^4+^ and AEs from ^125^I. Consequently, Ti^3+^ stretched the O—H bond of the absorbed H_2_O to decrease its bond energy. Finally, upon irradiation of γ‐rays emitted by ^125^I, the radiolysis of activated H_2_O will occur more easily, leading to an enhanced generation of •OH. Material characterization and DFT simulation confirmed the existence and function of the constructed active sites. In vitro and in vivo data further verified an enhanced curative effect of CIRT induced by the active sites. Compared with previous researches on nanocatalysts, this paper presented two major differences. First, the mechanism of the generation of ·OH in this research is different. In previous researches,^[^ ^42^, ^43^ ^]^ ·OH is usually generated from the reaction between H_2_O and photoinduced holes. While in this research, ·OH comes from the reaction between γ‐rays and H_2_O that is activated by AEs‐constructed active sites. Second, the excitation source of nanocatalysts in this research is different. Previous researches usually use an external excitation source like UV or vis, of which the penetration depth in biological tissue is only millimeter‐sized.^[^ ^44^, ^45^ ^]^ In this research, we solve this problem by using ^125^I as an internal excitation source. Hence, our strategy by using radionuclides to construct active sites in nanocatalysts will bring more ideas and chances for the applications of nanocatalysts. Moreover, we believe that this research will introduce new perspectives to the design of biomaterials and provide more opportunities for cancer therapy.

## Conflict of Interest

The authors declare no conflict of interest.

## Supporting information

Supporting Information
