# Supplementary material for: Auger Electrons Constructed Active Sites on Nanocatalysts for Catalytic Internal Radiotherapy
Source: Adv Sci (Weinh). 2020 Apr 6;7(10):1903585. doi: 10.1002/advs.201903585 (PMC7237849; doi:10.1002/advs.201903585)
Supplement: Supplementary file 1 — Supporting Information [file ADVS-7-1903585-s001.pdf]

Copyright WILEY-VCH Verlag GmbH & Co. KGaA, 69469 Weinheim, Germany, 2020.

ADVANCED SCIENCE
Open Access

Supporting Information

for *Adv. Sci.*, DOI: 10.1002/advs.201903585

Auger Electrons Constructed Active Sites on Nanocatalysts for Catalytic Internal Radiotherapy

Weiwei Su, Han Wang, Tao Wang, Xiao Li, Zhongmin Tang, Shuai Zhao, Meng Zhang, Danni Li, Xingwu Jiang, Teng Gong, Wei Yang, Changjing Zuo, Yelin Wu,* and Wenbo Bu**

Copyright WILEY-VCH Verlag GmbH & Co. KGaA, 69469 Weinheim, Germany, 2020.

Supporting Information

Auger Electrons Constructed Active Sites on Nanocatalysts for Catalytic Internal Radiotherapy

Weiwei Su, Han Wang, Tao Wang, Xiao Li, Zhongmin Tang, Shuai Zhao, Meng Zhang, Danni Li, Xingwu Jiang, Teng Gong, Wei Yang, Changjing Zuo, Yelin Wu,* and Wenbo Bu**

((Optional Dedication))

Dr. W. Su, Dr. T. Wang, Dr. X. Li, Dr. S. Zhao, Dr. D. Li, Prof. C. Zuo
Department of Nuclear Medicine, Changhai Hospital, Naval Medical University, Shanghai 200433 (P.R. China)
E-mail: cjzuo@smmu.edu.cn
Dr. H. Wang, Dr. Z. Tang, Dr. M. Zhang, Prof. W. Bu
State Key Laboratory of High Performance Ceramics and Superfine Microstructures, Shanghai Institute of Ceramics, Chinese Academy of Sciences, Shanghai 200050 (P. R. China)
Dr. T. Gong, Dr. W. Yang, Prof. W. Bu
Shanghai Key Laboratory of Green Chemistry and Chemical Processes, School of Chemistry and Molecular Engineering, East China Normal University, Shanghai 200062 (P. R. China)
E-mail: wbbu@chem.ecnu.edu.cn
Dr. X. Jiang, Dr. Y. Wu
Tongji University Cancer Center, Shanghai Tenth People's Hospital, Tongji University School of Medicine, Shanghai 200072 (P.R. China)
E-mail: sk_wuyelin@tongji.edu.cn
Dr. H. Wang, Dr. Z. Tang, Dr. M. Zhang
University of Chinese Academy of Sciences, Beijing 100049 (P.R. China)

Keywords: I-125, Nanocatalysts, Titanium dioxide, Active sites, Auger Electrons, Internal radiotherapy

1. Supplementary Methods

Materials. Chemical agents: Oleic acid, oleylamine, 1,3,4,6-Tetrachloro-3α, 6α-diphenylglycoluril (Iodogen), dichloromethane, 1-(3-dimethylaminopropyl)-3-ethylcarbodiimide (EDC) and N-hydroxysuccinimide (NHS) were purchased from Sigma-Aldrich Chemical Co., Ltd.. Tetrabutyl titanate, ethyl alcohol, cyclohexane, diethylene glycol, sodium citrate and tyramine were obtained from Adamas Reagent, Ltd.. $Na^{125}I$ solution was provided by Shanghai Xinke Pharmaceutical Co., Ltd.. All above agents were used without further purification.

Bioreagents: Phosphate buffered saline (PBS), dulbecco's modified eagle medium (DMEM) and fetal bovine serum (FBS) were obtained from GIBCO (Grand Island, New York, USA). Deionized (DI) water was supplied by a Milli-Q system (Health Force Bio-meditech Holdings Ltd.). Cell counting kit-8 (CCK-8) and hydroxyphenyl fluorescein (HPF) fluorescent probe of •OH were bought from Shanghai Yeasen Biotech Co., Ltd.. Moreover, 4% Paraformaldehyde, 2.5% glutaraldehyde, and antibodies for western blotting, immunofluorescence assays and pathological immunohistochemistry were offered by Wuhan servicebio technology Co., Ltd.. Giemsa stain was acquired from Sinopharm Chemical Reagent Co., Ltd..

Synthesis of TiO_2-tyr. The procedure was based on a typical solvothermal method. Firstly, Oleic acid-coated TiO_2 NPs (TiO_2-OA) were prepared. Tetrabutyl titanate (5 mmol, 1.7 g), oleic acid (25 mmol, 7.06 g), oleylamine (25 mmol, 6.69 g) and ethyl alcohol (3.20 g) were mixed homogeneously and stirred for 10 min. Ethyl alcohol (15.15 g) and DI water (0.8 g) were then added for another 5 min stirring. The mixture was then transferred to a hydrothermal synthesis reactor, and maintained at 180 ℃ for 18 h. After washed 3 times with ethanol and cyclohexane, the product was dispersed in cyclohexane (20 mL).

Secondly, to produce citric acid-coated TiO_2 NPs (TiO_2-COOH), diethylene glycol (30 mL) and sodium citrate (4 mmol) were mixed with the above solution (5 mL), heated to 160 □ and stirred for 3 h under argon (Ar) gas protection. After the reaction system was cooled down, the superfluous diethylene glycol was removed by centrifugation at 19,000 revolutions per

minute (rpm) for 30 min. The obtained sediment was redissolved in DI waster for a second centrifugation at 19,000 rpm for 30 min, and the collection was redispersed in ethyl alcohol for a third centrifugation at 19,000 rpm for 10 min. The mixture was clear and transparent after redispersed in 10 ml DI water.

Finally, for tyramine modification of TiO_2 NPs (TiO_2-tyr), the above solution (10 mL) was added with EDC·HCl (0.1 mmol) and NHS (0.3 mmol) and fully stirred for 30 min, and then reacted with tyramine (1 mmol) in the dark overnight. The final product of TiO_2-tyr was rinsed for several times and re-suspended in DI water for further use.

Characterization of TiO_2 NPs. Transmission electron microscopy (TEM) morphology of the TiO_2 NPs was confirmed by a FEI Tecnai G2 F30. X-ray diffraction (XRD) patterns were performed on a Rigaku D/MAX-2250V diffractometer at Cu Kα (λ = 0.154056 nm) with the scanning speed of 20° min^{-1} and range from 20° to 80°. Characteristic UV-Vis absorption spectra of TiO_2 NPs after the surface modification of oleic acid (TiO_2-OA), citric acid (TiO_2-COOH) and tyramine (TiO_2-tyr) were determined by a UV-Vis spectrophotometer (UV-3600, Shimadzu, Japan). Fourier transform infrared (FT-IR) spectra was obtained from a Bruker TENSOR II FTIR Spectrometer (Bruker Corporation) using KBr pellets. Zeta potential and the hydrodynamic size of TiO_2 NPs with different modifications were measured by Nanotrac Wave II Q Nanoparticle Size Analyzer (Microtrac Inc., USA). X-ray photoelectron spectra (XPS) were recorded using a VG ESCALAB 250Xi instrument.

^{125}I labeling rate and stability tests. ^{125}I was labeled *via* a standard Iodogen-catalyzed method:[1] Free ^{125}I (Na^{125}I solution in PBS) was quickly added into two Eppendorf (EP) tubes with each coated with 50 μg Iodogen, and slowly oscillated for 15 min. Next, TiO_2-tyr (144 ug, 7.3 mg mL^{-1}) was added into the tubes followed with another 30 min reaction at room temperature. The tubes were manually shook every 15 min for sufficient reaction.

The labeling rate and stability were monitored by radio thin layer chromatography (radio-TLC, Mini-scan, B-MS-1000F, Eckert & Ziegler radiopharma, Inc. MA, USA) with a *γ*-

detector, and Whatman chromatography paper and 0.9% NaCl solution were applied as the stationary and mobile phase, respectively. The *in vitro* stability of the labeling production ^{125}I-TiO_2 was tested by incubating with PBS solution containing 0.1% (v %) FBS at 37 °C for 24 h.

Calculated simulations. The first-principles calculations were carried out based on density functional theory. The generalized gradient approximation (GGA) of Perdew-Burke-Ernzerhof (PBE) was used to describe the exchange and correlation terms, and the Projector-Augmented Wave (PAW) pseudopotential method was also adopted in our system. The TiO_2 surfaces with the Ti^{3+} and Ti^{4+} structures have been established. In addition, the cut-off energy is set as 400 eV, and the Brillouin zone is sampled by a 3 × 3 × 1 k-grid. Finally, the optimization of the surface structure with adsorbed H_2O was continued until forces on each atom were smaller than 0.03 eV/Å.

***In vitro* cytotoxicity of TiO_2 NPs by CCK-8.** The anthropogenic pancreatic cancer (SW1990) cells were purchased from Shanghai Institute of Biochemistry and Cell Biology, Chinese Academy of Sciences, and cultured in DMEM with 10% FBS, at the condition of 37 °C in humidified atmosphere with 5% CO_2.

The cell toxicity was evaluated through CCK-8 assay. Firstly, SW1990 cells were seeded on four 96-well plates (2×10^4 cells per well), each formed a matrix with 9 columns (concentration gradient of NPs) multiplying 3 lines (three replication wells). The cells were adhesive 24 h later, and the culture medium was changed to TiO_2 solution in DMEM, with the concentration of TiO_2 ranging from 0 μg mL^{-1} and 7.8 μg mL^{-1} doubled to 1000 μg mL^{-1}. After 24 h and 48 h co-incubation, respectively, CCK-8 solution (15 μL) was added to each well and stained for 3 h. Finally, the iMark™ microplate reader (Bio-Rad, Hercules, CA, USA) was applied to test the absorbance value at 450 nm, and the cell viability was obtained by calculating the absorbance percentage of each group relative to that of the control group.

Cellular uptake of TiO_2 NPs. The intracellular uptake and distribution of TiO_2 NPs were observed by bio-TEM scanning. In detail, adherent cells that were pre-seeded in six-well plate (about 10^5 cells per well) were treated with TiO_2 (2,400 μg) in DMEM solution for 30 min, 1.5 h, 3 h or 5 h, respectively. Next, the cells were trypsinized, centrifugated and collected, followed with fixed by electron microscopy fixative (2.5% glutaraldehyde) at 4 ℃ for 3 h. Then, the cells were dehydrated with ethyl alcohol and acetone, osmosed with acetone and 812 embedding medium, embedded at 60 ℃ for 48 h and sliced into 60 nm-sick slices. After stained by uranium salts and lead salts for 15 min and dried overnight at room temperature, the slices were finally imaged under a bio-TEM system (Hitachi, HT7700, 80.0 kv).

Cell viability under ^{125}I-TiO_2 by CCK-8 assay. SW1990 cells were planked in 96-well plate (three replication wells, 2×10^4 cells per well) for adherent growth. The culture media was then replaced with DMEM containing free ^{125}I or ^{125}I-TiO_2 with varying dosages of ^{125}I (0, 100, 200, 400, 600, 800 and 1000 μCi mL^{-1}) for additional 24 h co-culture. Cell viability *via* CCK-8 assay was examined referred to above procedure of CCK-8 assay.

Cellular experiments. For *in vitro* therapeutic experiments except for clone formation assay, basic treatments of cells prior to the final assay tests were consistent: In detail, SW1990 cells (three groups, three duplications for each group) were seeded onto six-well culture plates, except that the cells for •OH test by fluorescence assay were grown on the preset cell culture slides placed in the wells. After adherence, the medium was substituted by DMEM, or DMEM containing $Na^{125}I$ or ^{125}I-TiO_2 (600 μCi mL^{-1} of ^{125}I corresponding to 144 μg mL^{-1} of TiO_2). The treatment lasted for 24 h, then cells were trypsinized, centrifugated, collected and resuspended in PBS for western blotting assay, or fixed by 4% paraformaldehyde for immunofluorescence tests. Specially, for cellular •OH detection by fluorescence assay, the cells adhered to slices were rinsed by PBS for direct test under the microscope. The subsequently detailed testing process of above collected samples was depicted in the following.

Cell apoptosis analysis by TUNEL. The fixed SW1990 cells (about 2×10^6 cells per sample) were collected and smeared, permeabilized with Triton X-100 (0.5%, 75 μL) for 20 min, incubated with the TUNEL kit (TdT : dUTP = 1 : 9) for 2 h, counterstained with DAPI for 10 min, and finally mounted by antifade mounting medium. All of the slices were imaged by fluorescence microscope (Nikon Eclipse CI, Japan), with exciting light for DAPI and FITC at 355 nm and 480 nm, respectively. The positive rates were quantified by an Image-pro plus 6.0 (Media Cybernetics, Inc., Rockville, MD, USA) software.

Analysis of PCNA expression by immunofluorescence assay. The collected SW1990 cells (about 2×10^6 cells per sample) after different treatments were fixed, harvested, resuspended and smeared on slices. Next, cells were permeabilized with Triton X-100 (0.5%, 75 μL) for 30 min and blocked by bovine serum albumin (BSA, 3 %) for 30 min. Then, cells were incubated with primary antibody (PCNA, dilution 1 : 200) at 4 ℃ overnight and labeled with secondary antibody (Cy3-labeled Goat Anti-Mouse IgG (H+L), dilution 1 : 300) for 50 min in dark. After stained with DAPI for 10 min in dark, slices were mounted and prepared for imaging. The fluorescence microscope (Nikon Eclipse CI, Japan) was used to acquire fluorescence images (DAPI excited at 355 nm, PCNA excited at 535 nm). The average optical density (AOD) was measured using Image-pro plus 6.0 (Media Cybernetics, Inc., Rockville, MD, USA) software.

Cell colony formation assay. The adherent SW1990 cells in regular growth were trypsinized and resuspended in DMEM. After sorted into predetermined counts (200, 400, 1000 and 2000 cells) by a MoFlo XDP Cell Sorter (Beckman Coulter, USA) *via* a streaming sorting technique, the cells were reseeded into six-well plates for 24 h adherent growth. Later, the medium was replaced by DMEM containing ^{125}I or $^{125}I\text{-}TiO_2$, with incremented doses (0, 2.5, 5 and 7.5 μCi) of ^{125}I for treating increasing numbers of cells as above mentioned. The treatment lasted for 24 h, and then the treatment solution was discarded, and cells were continued for a regular cultivation in DMEM containing 10% FBS until megascopic cell

colony (with more than 50 cells) formed after 10 days. For colony staining, after the cells were fixed by 75% ethanol (1.5 mL per well) for 10 min, each well was added with 0.5% Giemsa (1.5 mL per well) for 10 min incubation at room temperature and then cleaned and dried. Cell colonies were counted and recorded, and the cell cloning efficiency (CE, percentage of colonies relative to the inoculated cells) and cell surviving fraction (SF, ratio of CE of therapeutic group to the control group) were calculated.

Western blotting analysis. The SW1990 cells (about 2×10^6 cells per sample) after treatment were trypsinized and collected. Cellular proteins were extracted after lysed by RIPA buffer (250 μL), and its concentration was calculated by BCA kit. The obtained protein samples were loaded into agarose gel, separated by SDS-polyacrylamide gel electrophoresis and transferred to polyvinylidene fluoride (PVDF) membranes (Millipore, USA). The membranes were blocked with 0.5% skimmed milk, and incubated with primary antibodies including Bax (1 : 750 dilution, Servicebio), Bcl-2 (1 : 20000 dilution, Abcam), Ki-67 (1 : 750 dilution, Servicebio) and GAPDH (1 : 45000 dilution, PTG). Next, the membranes were probed with secondary antibodies conjugated with HRP (dilution 1 : 3000, Servicebio). Finally, membranes were exposed to electrochemiluminescence (ECL) reagent kit (Servicebio) before developing and fixing process. Bands images were acquired *via* an Epson scanner (Perfection V 300, China).

Intracellular •OH evaluation under confocal microscopy. The SW1990 cells (2×10^6 cells per sample) that mounted on slides of different treatment groups were washed twice by PBS and stained with HPF probe for 40 min in dark. After rinsed again by PBS and dried, the cell slides were observed by a confocal fluorescence microscope (Olympus FV 1000; Olympus, Tokyo, Japan) under 488 nm excitation for HPF. The fluorescence intensity of positive staining of cells was measured by an Image J (Version 1. 8. 0, National Institutes of Health, USA) software to reflect the amount of •OH.

DNA DSBs detection by immunofluorescence assay. SW1990 cells (about 2 × 10^6 cells per sample) from different groups were fixed, harvested, smeared on coverslips, permeabilized by Triton X-100 (0.5%, 75 μL) and blocked with BSA (3%). Subsequently, slices were treated by the primary antibody (γ-H2AX, 1 : 400 dilution) at 4 °C overnight, followed by co-incubation with the secondary antibody (Cy3-labeled Goat Anti-Rabbit IgG (H+L), 1 : 400 dilution) for 50 min away from light. Next, the nucleus of cells was stained with DAPI for 10 min and the cell slices were mounted to avoid fluorescence quenching. At last, the slices were observed under fluorescence microscope (Nikon Eclipse CI, Japan), and the blue and red fluorescence was excited at 355 nm and 535 nm, respectively. For quantitative analysis, the red foci of γ-H2AX were counted and the average number in each group was calculated and compared.

Animal modal. All animal experiments were performed according to the guideline of Experimental Animal Ethics Committee of the Second Military Medical University (Approval Number, **20181101087**). Balb/c nude mice (5 weeks, female) were provided by Shanghai Jihui Biological Technology Co. Ltd.. To establish xenograft tumor model, totally 5 × 10^6 SW1990 cells suspended in PBS (50 μL) were subcutaneously injected into the root of right posterior limb of each mouse. Once the tumors grew to approximately 100 cm^3, the mice in good health condition were selected and divided randomly into three groups for subsequent *in vivo* treatment.

***In vivo* SPECT/CT imaging and therapeutic evaluation.** Mice in three groups (five mice per group) were intratumorally injected with 10 μL pure DMEM, or DMEM solution containing ^{125}I or ^{125}I-TiO_2 with 600 μCi of ^{125}I corresponding to 144 μg TiO_2. Considering the enough long half-life period (59.6 days) of ^{125}I with continuous emission of the decaying product, the mice were administered with the above drugs for only once during the whole treatment process. At the initial treatment, SPECT/CT scanning (Symbia T16, Siemens, Germany) for multiple time points (0.5 h, 2 h, 6 h, 12 h, 24 h, 48 h, 120 h, 192 h, 288 h) post-

injection (P.I.) was conducted. Afterwards, the mice were weighted, and tumors were measured (length and width, mm) and photographed within 20 days P.I.. The relative tumor volume ($V\ V_0^{-1}$) was given when the tumor volume ($V = \text{length} \times \text{width} \times \text{width} \times 2^{-1}$) was divided by the initial tumor volume (V_0) at 0 d P.I. Also, the survival status of mice in three groups (eight mice per group) was closely observed and recorded within 60 days P.I..

At 20 d P.I., the tumors from three randomly selected mice in each group were dissected and sliced for pathological examination, including Hematoxylin and Eosin (H&E) staining, TdT mediated dUTP nick end labeling (TUNEL) and immunohistochemical analysis of Ki-67 and TNF-α.

Statistical analysis. In this article, all original data of the experiments were directly applied for statistical analysis, and presented as mean ± standard deviation (s.d.) in this work. The sample size for statistical calculation in most experiments was n = 3, except that for counting *γ*-H2AX foci of cells (n = 10) and evaluating tumor growth of mice (n = 5). All data were tested for their normality and homogeneity of variance before determining the specific statistical method. Statistical differences between two groups were analyzed by unpaired Student's t test for data simultaneously conform to normal distribution and homogeneity of variance, or a Mann-Whitney U test would be performed. Statistical differences among three groups was tested by one-way ANOVA followed with LSD-t post-hoc test for normally distributed sets with equal variance, or Kruskal-Wallis 1-way ANOVA test for those with non-normal distribution. With a testing level of $\alpha = 0.05$, the two-sided P value less than 0.05 was considered as statistically significant. In a detailed presenting form, the symbol P was labeled with different number of asterisks (*) according to its actual value (*$P < 0.05$; **$P < 0.01$; ***$P < 0.001$). All the statistical processes were performed *via* SPSS 21.0 software (IBM Corp., Armonk, NY, USA).

2. Supplementary Figures

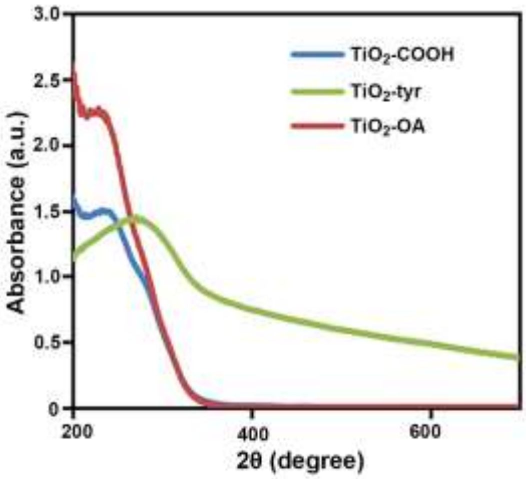

Figure S1. UV-Vis spectra of TiO_2-OA, TiO_2-COOH and TiO_2-tyr.

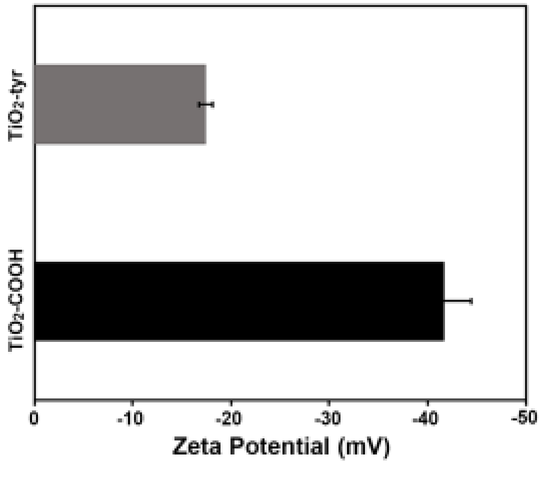

Figure S2. Zeta potentials of TiO_2-COOH and TiO_2-tyr.

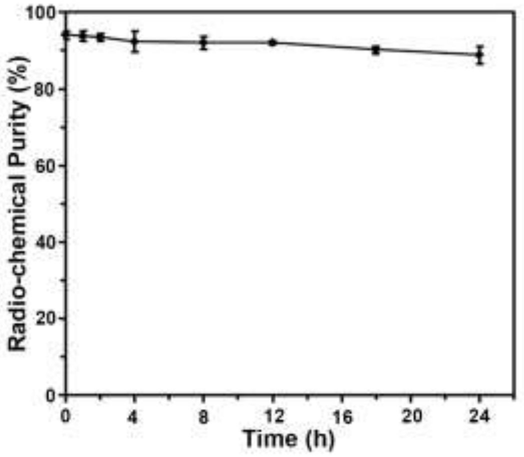

Figure S3. Radiolabeling stability of ^{125}I-TiO_2 within 24 h co-incubation in 0.1% FBS solution.

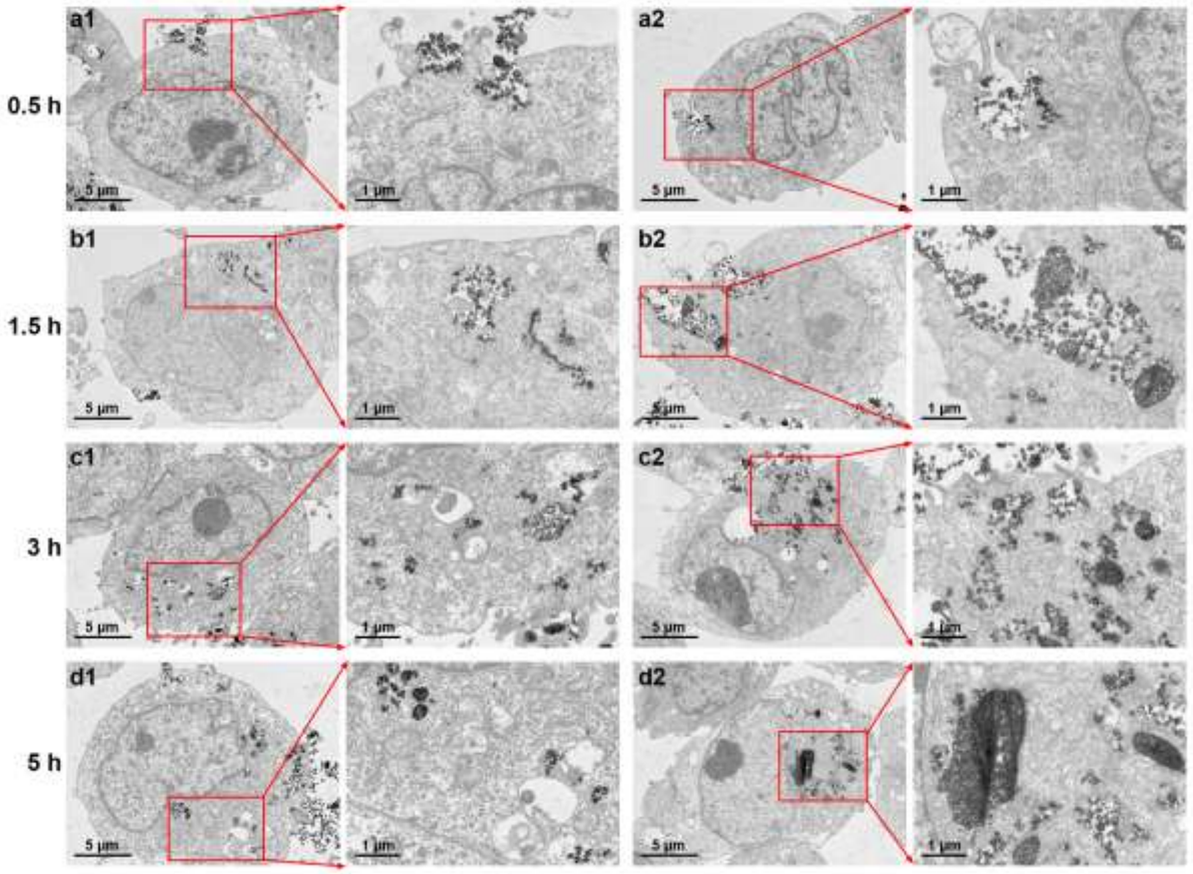

Figure S4. Bio-TEM images of SW1990 cells after incubated with TiO_2 NPs solution for 0.5 h (a1, a2), 1.5 h (b1, b2), 3 h (c1, c2) and 5 h (d1, d2), respectively.

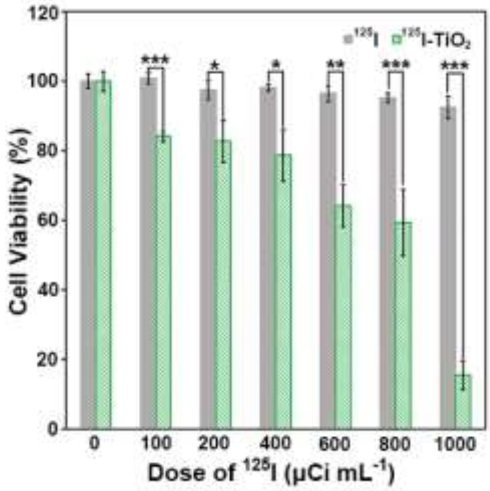

Figure S5. Relative viability of SW1990 cells treated with ^{125}I and ^{125}I-TiO_2 at incremental ^{125}I doses (n = 3, mean ± s.d., P = 0.0002, 0.0184, 0.0109, 0.0010, < 0.0001 and < 0.0001 under the dose of 100, 200, 400, 600, 800 and 1000 μCi $mL^{-1}$of ^{125}I, respectively. $*P < 0.05$, $**P < 0.01$, $***P < 0.001$). The significance of statistical differences of cell viability between ^{125}I and ^{125}I-TiO_2 groups under ^{125}I dose of 0, 100, 200, 400 and 600 μCi mL^{-1} was determined by unpaired Student's t test, and that under ^{125}I dose of 800 and 1000 μCi mL^{-1} was analyzed by Mann-Whitney U test.

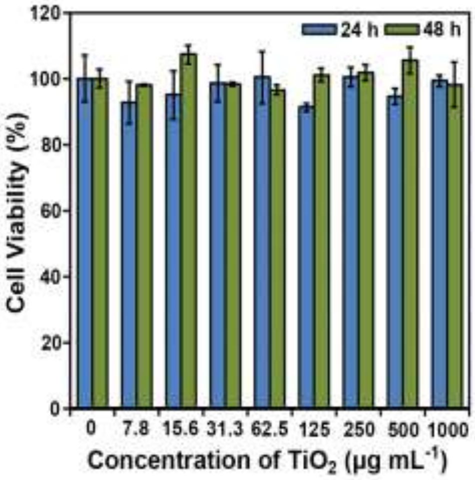

Figure S6. The relative viability of SW1990 cells after 24 h or 48 h co-incubation with different concentrations of TiO_2 (n = 3, mean ± s.d.).

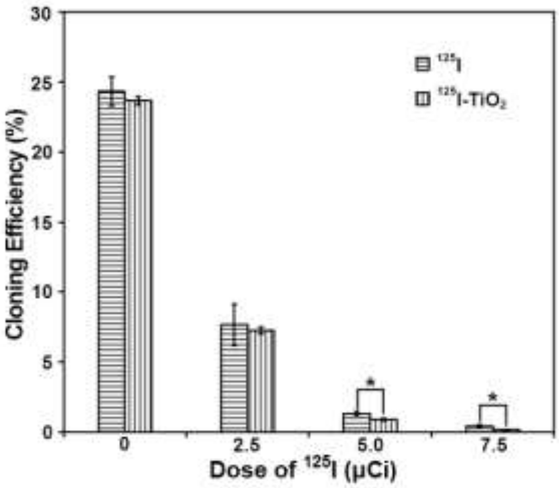

Figure S7. Calculation and comparison of cell cloning efficiency (n = 3, mean ± s.d., P = 0.0147 and 0.0463 under the ^{125}I dose of 5.0 μCi and 7.5 μCi. *P < 0.05). The significance of statistical differences of cell cloning efficiency between ^{125}I and ^{125}I-TiO_2 groups under ^{125}I dose of 5 μCi was evaluated by unpaired Student's t test, and that under ^{125}I dose of 0, 2.5 and 7.5 μCi was determined by Mann-Whitney U test.

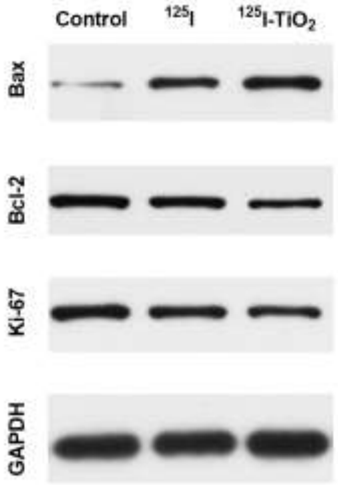

Figure S8. Western blotting analysis of cell proliferation- and apoptosis- related proteins after different treatments.

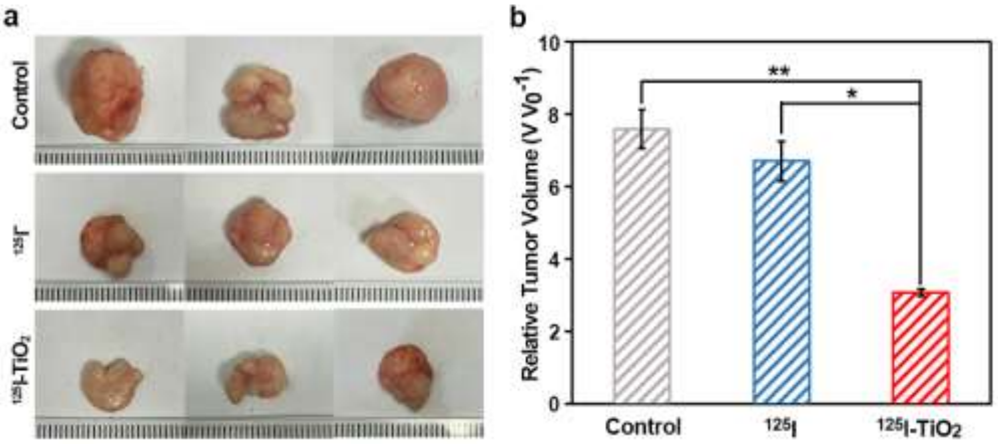

Figure S9. a) Representative photographs of tumors harvested from mice at day 20 after different treatments. b) Quantitative comparison of the relative volumes ($V\ V_0^{-1}$) of tumors collected from mice after 20 days' treatment. (n = 5, mean ± s.d., Kruskal-Wallis 1-way ANOVA test, P = 0.048 (^{125}I-TiO_2 *vs.* ^{125}I) and 0.001 (^{125}I-TiO_2 *vs.* Control), *P < 0.05, **P < 0.01).

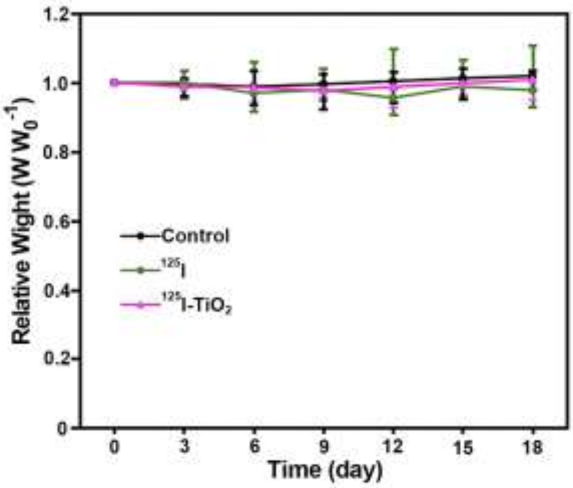

Figure S10. Time-dependent body weight change of mice within 20 days after different treatments (n = 5, mean ± s.d). The relative body weight ($W\ W_0^{-1}$) was given when the weight (W) measured at the given time was divided by the initial weight (W_0) at 0 d P.I..

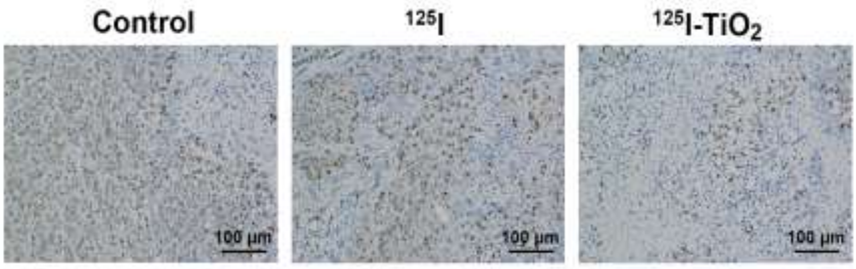

Figure S11. Representative immunohistochemistry images of Ki-67 staining for tumors collected at day 20 after different treatments (original magnification 200 ×).

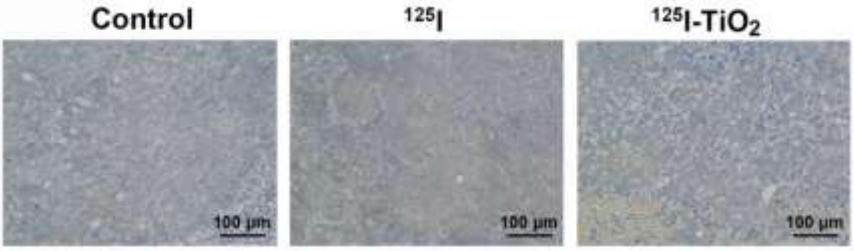

Figure S12. Representative immunohistochemistry images of TNF-α staining for tumors collected at day 20 after different treatments (original magnification 200 ×).

References

[1] G. Sun, T. Wang, X. Li, D. Li, Y. Peng, X. Wang, G. Jia, W. Su, C. Cheng, J. Yang, C. Zuo, *Adv. Healthc. Mater.* **2018**, *7*, 1800375.
